# Biomechanical properties of feline ventral abdominal wall and celiotomy closure techniques

**DOI:** 10.1111/vsu.12751

**Published:** 2017-11-18

**Authors:** Fernando S. Reina Rodriguez, Conor T. Buckley, Joshua Milgram, Barbara M. Kirby

**Affiliations:** ^1^ University College Dublin School of Veterinary Medicine Dublin Ireland; ^2^ Trinity College Dublin Centre for Bioengineering Dublin Ireland; ^3^ Koret School of Veterinary Medicine Hebrew University of Jerusalem Jerusalem Israel

## Abstract

**Objective:**

To compare biomechanical properties and mechanism of failure of 3 regions of ventral abdominal wall in cats by using 2 suture materials, 2 suture bite‐to‐stitch intervals (SBSI), and full‐thickness versus fascia‐only closure.

**Study Design:**

Randomized, cadaveric, ex vivo mechanical testing.

**Sample Population:**

16 adult cat cadavers, 3 samples per cat.

**Methods:**

Three regions of ventral abdominal wall were mechanically tested (N = 48 samples). Preumbilical, umbilical (U), and postumbilical (POU) regions were harvested by using a template. The thickness of the linea alba was recorded. Six samples without celiotomy served as controls. Twenty‐eight samples were randomized to SBSI (2 × 2 or 5 × 5 mm) and suture material (3‐0 polyglactin 910 or 3‐0 polydioxanone) for simple continuous celiotomy closure. Fourteen samples were randomized to full‐thickness or fascia‐only closure. Samples were tested by linear distraction; tensile strength and mechanism of failure were recorded. Effects of body weight, thickness of linea alba, anatomic region, SBSI, type of closure, and suture material were evaluated by mixed model linear analysis. Load to failure was compared between males and females, full‐thickness and fascia‐only closure by independent *t* test, with *P* < .05 considered statistically significant.

**Results:**

The POU region achieved lower loads to failure. Load to failure was greater in males compared with females. No difference was detected between full‐thickness and fascia‐only closure. Failure most commonly occurred by tearing of suture through tissues. Tissue failure with suture line loosening occurred mainly in the 5 × 5‐mm SBSI group.

**Conclusion:**

The POU region is biomechanically weak and may therefore be predisposed to incisional herniation.

## 1 INTRODUCTION

Ventral midline celiotomy is commonly performed in small animals. Long incisions are required for exploration and complex procedures,^1^ whereas short incisions in the caudal abdomen are used for ovariohysterectomy and cystotomy. Complications of ventral midline celiotomy in small animals include surgical site infection, peritonitis, wound dehiscence, and incisional herniation with or without eventration.^2^ Incisional dehiscence following ventral midline celiotomy may occur acutely or chronically, weeks to years after surgery.^3^ Although a 0.18% rate of incisional hernia has been reported in small animals following ventral midline celiotomy,^4^ this complication is likely under reported. Incisional complications were observed in 4% of patients in a retrospective study of 200 dogs and cats undergoing exploratory laparotomy, but the incidence of incisional herniation was not specifically reported.^1^ Incisional hernia was reported in 11 of 292 dogs (3.8%) and 8 of 74 cats (10.8%) in a retrospective study of types of hernias (C. Bellenger, personal communication, 1990). Major abdominal evisceration injuries were described in a retrospective study of 8 dogs and 4 cats, located at the incision for ovariohysterectomy in half of the dogs and all of the cats.^5^ Incisional hernias in domestic cats appear to occur more frequently after caudal ventral celiotomy.^5^ Although this likely reflects the high prevalence of ovariohysterectomy among abdominal procedure performed in this species, it is unknown whether anatomical differences between cranial and caudal ventral abdominal wall,^6^ different thickness along the linea alba, and/or different biomechanical properties of abdominal wall muscles^7^ could contribute to ventral incisional hernias in the caudoventral abdomen in domestic cats.

Guidelines for ventral midline celiotomy closure in small animals include recommendations for distance between suture bites and stitches, but these are based on clinical experience^4^, ^8^ and are not supported by biomechanical testing in cats. To the best of our knowledge, biomechanical studies have been performed only in dogs.^4^, ^9^ In these studies, the external leaf of rectus abdominis sheath has been identified as the primary strength‐holding layer for ventral paramedian incision closure in dogs.^9^ To the best of our knowledge, fascia‐only closure has not been previously reported in domestic cats. The force that the feline abdominal wall must resist to prevent wound dehiscence is unknown. Establishment of feline anatomical and biomechanical data may provide evidence to support recommendations for ventral midline celiotomy closure in cats.

Our study seeks (1) to compare the biomechanical properties and mechanism of failure of discrete segments of feline ventral abdominal wall and linea alba (preumbilical region [PU], umbilical region [U], and postumbilical region [POU]), (2) to compare the load to failure and mechanism of failure of celiotomy closure in discrete segments of feline ventral abdominal wall by using 3/0 polydioxanone and 3/0 polyglactin 910 and 2 suture‐bite‐stitch intervals (SBSI), and (3) to compare the load to failure and mechanism of failure of celiotomy closure in discrete segments of feline ventral abdominal wall using full‐thickness suture bites or fascia‐only suture bites in the closure. We hypothesized that (1) the POU region of feline ventral abdominal wall is biomechanically weak compared with the PU and U regions, (2) there is no biomechanical difference in celiotomy closure between the 2 suture materials tested, (3) there is no biomechanical difference between 2 SBSIs tested, and (4) there is no biomechanical difference between full‐thickness and fascia‐only suture bites in feline celiotomy closure.

## 2 MATERIALS AND METHODS

Full ethical approval was obtained from the institutional animal research ethics committee of the University College Dublin School of Veterinary Medicine, Dublin, Ireland.

### 2.1 Tissue collection and sample preparation

**Figure 1:**
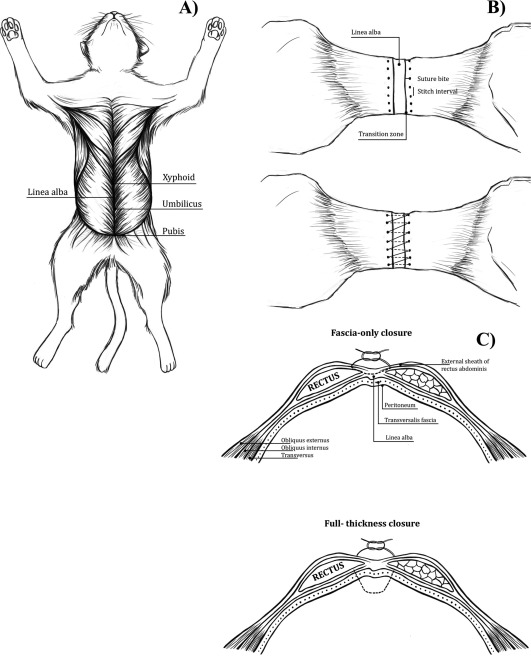
A, Cat in dorsal recumbency after skin removal from ventral abdomen. B, “Dog bone” shaped samples indicating suture bite and stitch interval. C, Diagram of needle penetration for full‐thickness and fascial‐only closure

Adult domestic cats euthanized for reasons unrelated to this study with no evidence of systemic or local disease affecting abdominal wall (eg, obesity, cachexia, recent abdominal surgery, abdominal trauma, or neoplasia) were donated. Cadavers were stored frozen at −80°C within 30 minutes after euthanasia and thawed at room temperature 24 hours prior to testing. Body weight (BW; kg), gender, and breed were recorded. With the cat in dorsal recumbency, hair was clipped and the abdominal wall was inspected for bruising, asymmetry, wounds, defects, or other evidence warranting exclusion. Skin and subcutaneous tissue were removed. Ventral abdominal wall was harvested by transverse incisions at caudal margin of xyphoid and cranial margin of pubis and longitudinal incisions 10 cm lateral to the linea alba on each side (Figure 1A). Immediately after harvest, the falciform ligament was removed, and the specimen was pinned to cardboard. Craniocaudal length was measured with a ruler and recorded.

**Figure 2:**
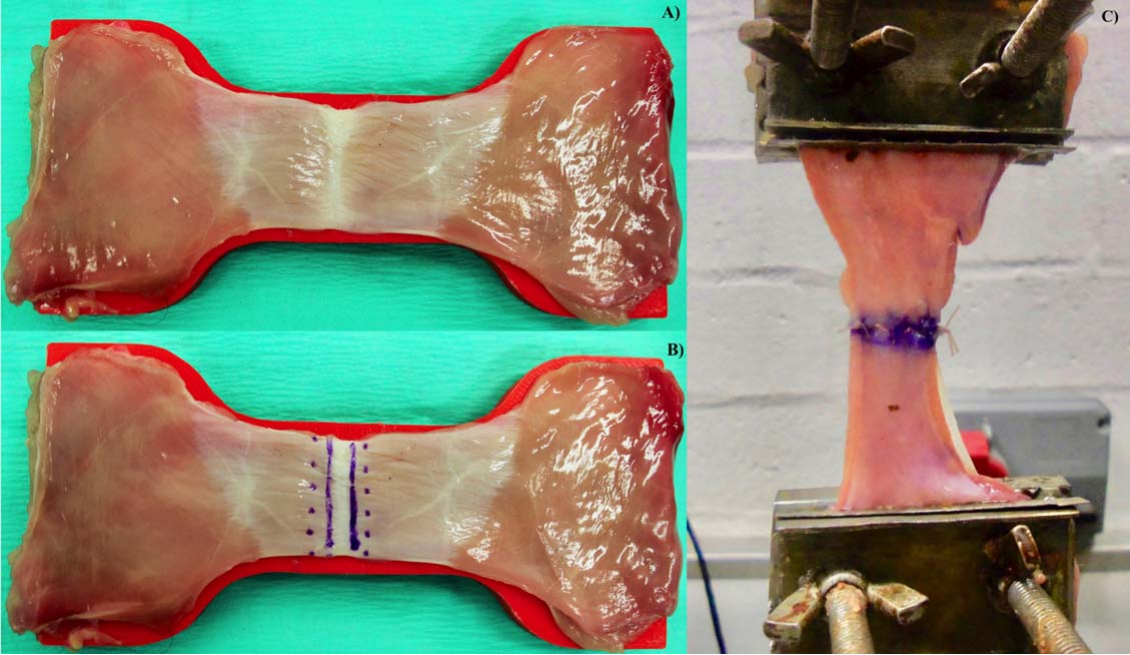
A, “Dog bone” shaped sample over template. B, 5 × 5 celiotomy sample. Points marked on external sheath of rectus abdominis to identify site of needle penetration during closure. The 2 parallel lines represent edges of the linea alba. C, Sample mounted in materials testing machine

Three regions of interest (ROI) were defined, PU, U, and POU, for each abdominal wall sample. One sample of each ROI was obtained from each cat by using a “dog bone” shaped template (polylactic acid) manufactured by 3D printer (Ultimaker 2+; Ultimaker BV, Geldermalsen, the Netherlands). The shape of the template was based on recommendations for tensile testing of materials with elastic properties.^10^ The template was 1.5 cm long with 5‐cm shoulders and 2.5‐cm width at its center (Figure 2A). Each ROI sample in the study was harvested with a scalpel blade after securing the template transversely to the body wall while centered directly over the linea alba. The sample at the linea alba was confirmed to measure 2.5 cm in width with digital calipers (Fisherbrand Traceable digital calipers; Thermo Fisher Scientific, Waltham, Massachusetts) immediately after harvest. In addition, the thickness of the linea alba was measured at the center of each sample with digital calipers and recorded. Care was taken to avoid compression of tissue with pressure of the calipers. Samples were immersed in a phosphate buffered saline bath to avoid desiccation and stored at 4°C prior to biomechanical testing performed within 6 hours after sample preparation.

Samples were randomly assigned to 3 groups (control, SBSI/suture material, and type of closure) by using computer software (SPSS Statistics, Version 20; IBM, Armonk, New York). Control samples were left intact. Celiotomy samples were bisected by scalpel on the linea alba. Celiotomy samples were closed by simple continuous suture secured with starting square knot of 5 throws and ending square knot of 6 throws.^11^ After completing knots, suture material was cut, leaving 3‐mm ends. Length of suture material used was calculated by adding lengths of remnants plus remaining length on needle (all measured with digital calipers) and subtracting from suture material length recorded on suture packet. Suture length to wound length ratio (SL:WL) was calculated as length of suture material used for closure divided by standardized wound length (2.5 cm).

SBSI/suture material group was divided into 4 subgroups based on SBSI and suture material used for celiotomy closure. SBSI was designated 2 × 2 or 5 × 5, indicating a distance of 2 mm or 5 mm, respectively, from suture bite to lateral edge of the linea alba (grossly identified as the junction between the linea alba and the external leaf of rectus abdominis muscle sheath) and a distance of 2 mm or 5 mm, respectively, between suture bites (Figure 1B). Distance from suture bite to the lateral edge of the linea alba and between suture bites was standardized and marked on each sample by using hectograph paper, a marker, and a ruler for accurate needle penetration and suture spacing. A template was drawn with lateral edges of the linea alba marked as solid lines and points marking distance (mm) between suture bites and distance (mm) from lateral edges of the linea alba marked for needle penetration for SBSI (Figure 2B). Celiotomy was closed by using either polyglactin 910 USP 3‐0 (Vicryl; Ethicon, Somerville, New Jersey) or polydioxanone USP 3‐0 (PDS II; Ethicon) on a 26‐mm SH taper needle. In “type of closure” group, celiotomy was closed by using simple continuous 2 × 2 SBSI and polydioxanone USP 3‐0 on a 26‐mm SH taper needle with full‐thickness or fascia‐only tissue bites (Figure 1C).

### 2.2 Biomechanical testing

Samples were mounted in a testing machine (MTM; Zwick Roell Z005; Zwick/Roell GmbH, Ulm, Germany) equipped with a 100 N load cell. Sample shoulders were secured with custom‐made clamps attached to stationary and moving anvils of the MTM, equidistant from the linea alba and oriented perpendicular to the direction of distraction (Figure 2C). Each clamp consisted of two 5 × 5‐cm metal plates with cheese grater blades secured to their inner surfaces and compressed the sample shoulders by tightening 2 screws 3 cm apart. Rotation of the sample in the grips prior to testing allowed uniform distribution of load across the sample width.

Samples were preloaded to 1 N and then tested under vertical displacement uniaxial loading at 0.5 mm/second to failure. Strain (%), tissue elongation (mm), and load to failure (N) were recorded for each test. Stress was calculated using the formula $\sigma =\;\frac{F}{A}$; where σ represents stress, *F* is applied load (N), and *A* (mm^2^) is initial cross sectional area calculated by multiplying the width (25 mm) and thickness of each sample. Mechanism of failure was recorded by digital camera (VG‐160; Olympus, Shinjuku, Tokyo, Japan). All tests were performed and interpreted by a single investigator.

Load to failure was defined as the maximum force (N) recorded before a sharp decline on load displacement curve followed by automatic termination of test. Test data were transferred automatically by computer software to an Excel 2010 document (Microsoft, Redmond, Washington). Mechanism of failure was evaluated and classified after completion of all biomechanical tests.

### 2.3 Statistical analysis

Data were analyzed in statistical software (SPSS Statistics, Version 20; IBM). A test for normality was performed for all variables. Descriptive statistics were performed to determine mean BW, thickness of linea alba, and load to failure. Spearman's rank correlation coefficient was calculated to evaluate correlation between BW, gender, and thickness of linea alba. Mixed model linear analysis was performed to evaluate effect of suture material, SBSI, type of closure, and anatomical location (repeat measures/fixed effects) on load to failure (dependent variable). BW, gender, and thickness of linea alba were subjects/random effects in the statistical model. Stepwise regression analysis was performed to remove nonsignificant variables from the statistical model; *P* < .05 was considered statistically significant. One‐way ANOVA for the final statistical model was performed to compare mean load to failure between groups, followed by post hoc analysis. An independent *t* test was performed to compare mean load to failure between full‐thickness and fascia‐only closure and load to failure for samples obtained from male and female cats. *P* < .05 was considered significant.

## 3 RESULTS

Eighteen adult cat cadavers were evaluated for inclusion in the study. Two cats were excluded, 1 with evidence of abdominal trauma and 1 with a large subcutaneous mass firmly adhered to and invading the ventral abdominal wall. Sixteen cats (N = 16) were included in the study. All specimens were abandoned cats donated from rescue centers. Information provided from the rescue centers was limited to estimated age (range, 2‐8 years) and date of euthanasia. There were 7 females (3 entire, 4 neutered) and 9 males (5 entire, 4 neutered). Breeds included 9 domestic shorthaired cats, 6 domestic longhaired cats, and 1 Abyssinian. The mean ± SD BW was 3.41 ± 1.16 kg (range, 1.30‐5.28).

Four female cats used in our study had undergone ovariohysterectomy. We did not observe evidence of previous surgical incision, suture material, scar, or adhesions on inspection of the ventral abdominal wall prior to harvesting the samples. We concluded that either a previous celiotomy incision was completely healed without visible evidence of incision or a flank approach had been used. These specimens were included in the study.

### 3.1 Thickness of the linea alba

**Figure 3:**
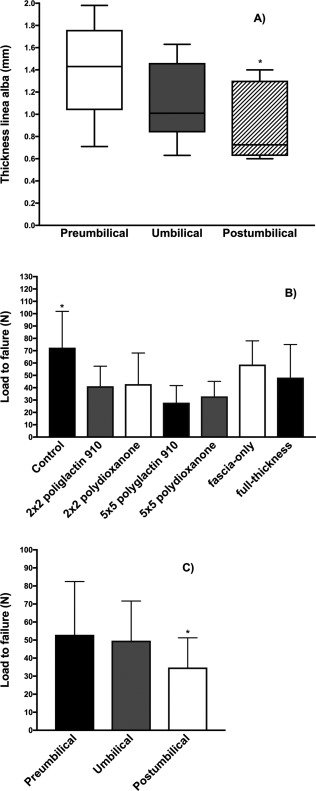
A, Thickness of the linea alba (mm) per region of interest (ROI). B, Load to failure (N) for control and celiotomy samples. C, Load to failure per ROI. Results are mean ± SD. *P < .05.

The thickness of the linea alba (mean ± SD) ranged from 0.6 to 1.98 mm (1.13 ± 0.40). The thickness ranged ranged from 0.71 to 1.98 mm (1.39 ± 0.4) in the PU region, 0.63 to 1.63 mm (1.08 ± 0.33) in the U region, and 0.6 to 1.4 mm (0.91 ± 0.32) in the POU region (Figure 3A). This thickness decreased gradually from cranial to caudal in all cats (*P* = .002), with thicker samples obtained from the PU region compared with those from the POU region, except in specimen 2, in which the thickness was identical in the PU and U regions. There was a moderate correlation between BW and thickness of the linea alba (*R* = 0.427, *P* = .02). No correlation was observed between gender and thickness of the linea alba (*P* = .179) or between gender and BW (*P* = .06).

### 3.2 Group distribution

Forty‐eight samples (N = 48) were tested biomechanically (3 samples per cat). Six samples from 2 cats (n = 6) served as controls, with 2 samples from each ROI. Forty‐two samples were obtained from the remaining 14 cats (14 samples from each ROI). Twenty‐eight of these 42 samples (n = 28) were allocated to the SBSI/suture material group (n = 28), with 7 samples (n = 7) in each subgroup, depending on suture material and SBSI used for celiotomy closure. Fourteen samples (n = 14) were allocated to type of closure group (n = 14), with 7 samples (n = 7) undergoing full‐thickness closure and the remaining samples (n = 7) undergoing fascia‐only closure. The number of samples from each ROI was variable between groups due to randomization.

### 3.3 Load to failure

SL:WL was >4 in all specimens. Load to failure (mean ± SD) ranged from 11.60 to 121.35 N (45.34 ± 23.81) among the 48 samples. Load to failure for control samples ranged from 33.38 to 121.35 N (68.88 ± 3.51) and was consistently highest in the PU region, lower in the U region, and lowest in the POU region. Load to failure (N) was 37.93 ± 5.27 for samples closed with polydioxanone, 34.55 ± 4.29 for samples closed with polyglactin 910, 42.05 ± 5.45 with a 2 × 2 SBSI, and 3.43 ± 3.41 with a 5 × 5 SBSI. Load to failure (N) was 48.18 ± 1.15 for full‐thickness closure samples, 58.73 ± 7.30 for fascia‐only closure samples, 52.64 ± 5.19 for samples obtained from males, and 35.95 ± 3.25 for samples obtained from females. Load to failure was not affected by suture material (*P* = .488), SBSI (*P* = .07), or type of closure (*P* = .533). Mean load to failure did not differ between suture material and SBSI groups (*P* = .082). Load to failure for gender, including neutering status, did not show significant differences between males and females (*P* = .102). However, an independent *t* test comparing load to failure between males and females without considering neutering status showed lower load to failure in females compared with males (*P* = .024). No significant differences were observed in load to failure of samples obtained from the 4 female neutered cats included in the study. Data are summarized in Table 1.

**Table 1:** Data from 48 samples^a^

				Thickness linea alba (mm)			Load to failure (N)			Strain to failure (%)			Stress at failure (N/mm^2^)			SL:WL		
Group		Specimen number	Body weight (kg)	*PU*	*U*	*POU*	*PU*	*U*	*POU*	*PU*	*U*	*POU*	*PU*	*U*	*POU*	*PU*	*U*	*POU*
Control (n = 6)		1	2.50	1.09	0.98	0.64	74.08	55.29	33.38	116.25	125.44	27.87	2.72	2.26	2.09			
		7	5.28	1.41	0.90	0.68	121.35	82.85	67.65	55.31	76.11	64.32	3.44	3.68	3.98			
Suture material SBSI	2 × 2 Polyglactin 910 (n = 7)	2	2.24		0.89			34.57			93.15			1.55			8.2	
		3	2.80	1.21			38.14			24.84			1.26			11.6		
		5	2.58	1.02			23.32			99.91			0.91			9.6		
		9	1.30	0.85			24.76			56.65			1.17			8.0		
		10	2.46	1.26			67.73			93.69			2.15			9.7		
		11	5.20			1.37			43.54			121			1.27			11.2
		12	4.36	1.70			56.19			35.86			1.32			7.4		
	2 × 2 Polydioxanone (n = 7)	2	2.24			0.74			47.50			78.64			2.57			7.6
		4	3.94		0.63	0.62		32.78	20.24		152.43	56.24		2.08	1.31		6.8	6.8
		5	2.58			0.60			23.88			45.02			1.59			7.5
		6	4.50	1.45			76.06			86.65			2.10			8.4		
		8	2.52	1.55			21.55			26.03			0.56			7.7		
		11	5.50		1.50			78.44			144.74			2.09			11.3	
	5 × 5 Polyglactin 910 (n = 7)	2	2.24	0.89			43.33			42.64			1.95			4.3		
		3	2.8		0.82	0.70		22.20	14.74		35.26	20.28		1.08	0.84		5.2	5.7
		4	3.94	0.71			24.84			24.81			1.40			6.4		
		5	2.58		0.91			30.37			142.00			1.34			6.1	
		9	1.30			0.60			11.60			34.56			0.77			7.4
		10	2.46		1.12			48.34			47.77			1.73			6.7	
	5 × 5 Polydioxanone (n = 7)	6	4.50		1.04	0.71		43.93	27.31		48.61	109.9		1.69	1.54		6.2	4.2
		8	2.52		0.75	0.62		22.96	17.57		54.90	94.83		1.22	1.13		4.3	4.8
		9	1.30		0.66			28.24			63.38			1.71			6.3	
		10	2.46			1			39.31			62.14			1.57			5.7
		11	5.20	1.98			51.28			50.65			1.04			6.6		
Type of closure	Full thickness (n = 7)	12	4.36			1.40			46.10			30.10			1.32			
		13	4.44			1.39			28.60			18.44			0.82			
		14	3.70	1.78	1.52		19.58	54.29		1.96	12.07		0.45	1.43				
		15	2.80	1.45		1.25	97.60		27.89	11.28		6.12	2.69		0.89			
		16	3.94			0.99			63.24			3.51			2.55			
	Fascia only (n = 7)	12	4.36		1.63			59.39			51.29			1.46				
		13	4.44	1.94	1.51		65.75	83.94		23.84	59.10		1.36	2.22				
		14	3.70			1.32			43.95			4.14			1.33			
		15	2.80		1.35			81.03			11.97			2.40				
		16	3.94	1.93	1.19		41.26	35.79		5.12	2.96		0.86	1.20				

POU, postumbilical; PU, preumbilical; SBSI, suture bite to stitch interval; SL:WL, suture length to wound length ratio; U, umbilical.

a Empty cell indicates data not recorded.

In the mixed model, no effect of suture material, SBSI, or type of closure was observed in load to failure (Figure 3B). When these variables were removed from the statistical model (stepwise regression analysis), anatomical location showed significance as a dependent variable with decreased load to failure in POU region samples (*P* = .049) compared with PU (*P* = .864) and U (*P* = .064) regions. One‐way ANOVA for anatomical location excluding suture material, SBSI, and type of closure showed decreased load to failure of the POU region (*P* = .009) compared with the PU and the U regions (Figure 3C). No statistical differences were observed between the PU and U regions (*P* = .34). BW contributed to 33.4% of variability in load to failure, and thickness of linea alba contributed to 35.4%.

### 3.4 Type of failure

**Figure 4:**
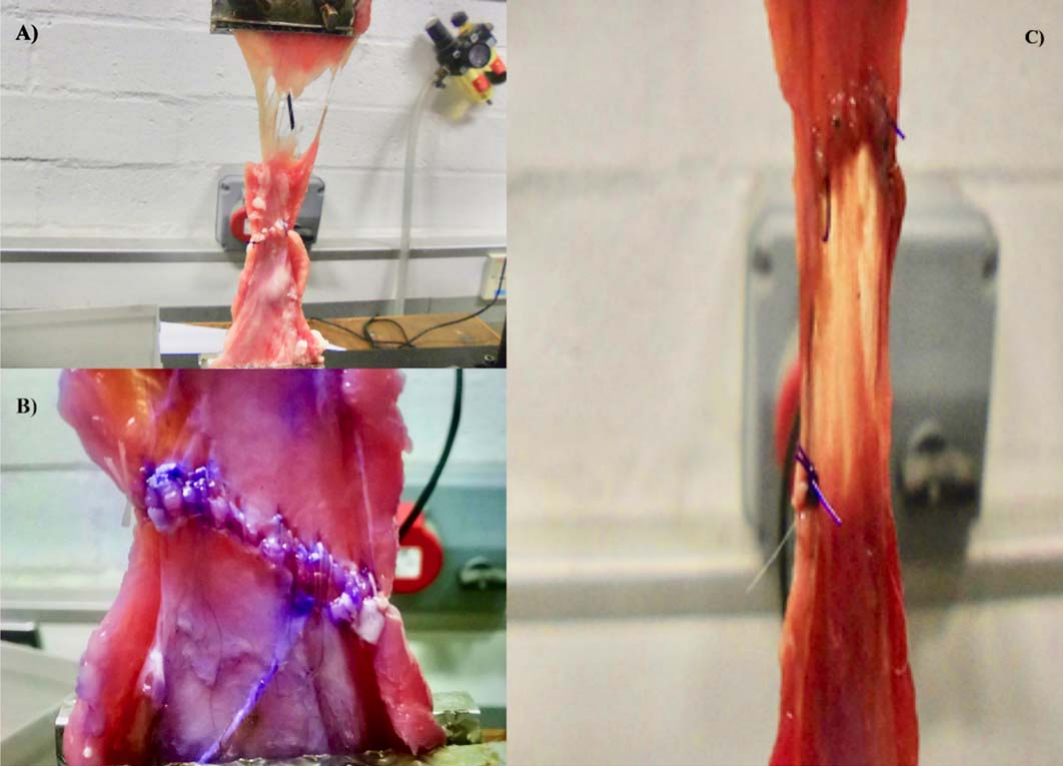
A, Failure distant from celiotomy closure in a 2 × 2 polydioxanone sample. B, Failure by muscle/fascial tearing in a 2 × 2 polyglactin 910 sample. C, Failure by loosening of suture line with intact knots

Type of failure was classified as (1) body wall failure distant from linea alba or celiotomy site, (2) failure of body wall by linear tears in muscle or fascia perpendicular to linea alba at site of suture material penetration(s) with suture line in celiotomy remaining intact, and (3) tissue failure at suture line without linear tears with loosening of suture line resulting in gap between incised wound edges and with intact knots. All control group samples (n = 6) failed by muscle tearing distant from the linea alba (Figure 4A). Two 2 × 2 SBSI samples (1 polydioxanone and 1 polyglactin 910), one 5 × 5 polyglactin 910 sample, and 3 fascia‐only closure samples failed by muscle tearing distant from the linea alba, similarly to control samples. Linear tears in muscle or fascia at points of suture penetration occurred in 5 of seven 2 × 2 polyglactin 910 samples, 6 of seven 2 × 2 polydioxanone samples, 3 of seven 5 × 5 polydioxanone samples, 4 of 7 fascia‐only closure samples, and all 7 full‐thickness closure samples (Figure 4B). Tissue failure without linear tears with loosening of suture line with knots intact occurred in 1 of seven 2 × 2 polyglactin 910 samples, 6 of seven 5 × 5 polyglactin 910 samples, and 4 of seven 5 × 5 polydioxanone samples (Figure 4C). Failure by muscle or fascial tearing was most commonly observed in the 2 × 2 SBSI samples, whereas failure by loosening of suture line occurred mainly in 5 × 5 SBSI samples, independent of suture material used for closure.

## 4 DISCUSSION

The main findings of our study are that (1) the POU region of the feline ventral abdominal wall was biomechanically weaker than the PU and U regions, (2) the thickness of the linea alba progressively decreased from cranial to caudal, (3) no difference was detected between incisions closed with polydiaxanone or polyglactin 910 suture materials or between full‐thickness and fascia‐only closures, and (4) the only difference identified between incisions consisting of small versus large bites consisted of tissue failure in which suture loosening without fascial tears was observed more frequently in the large bite group.

In a mixed model linear analysis adjusted for BW and linea alba thickness, the POU region sustained lower strain to failure, supporting our hypothesis that the POU region is biomechanically weaker than the PU and U regions. In dogs, the internal leaf of rectus abdominis sheath disappears, and the aponeurosis of the internal abdominal oblique muscle joins the external leaf of the rectus abdominis sheath. This sheath is covered dorsally only by a thin continuation of transversalis fascia and by the peritoneum in the caudal third of the abdominal wall.^6^ Similar anatomical findings in the cat may explain the lower strain to failure for the POU region observed in our study. Different characteristics and intrinsic properties of abdominal wall muscles have been described experimentally in dogs.^7^ Similarities in domestic cats could contribute to differences observed during tensile tests. To the best of our knowledge, this is the first study reporting the tensile strength of the feline ventral abdominal wall. In control samples, the POU region reached lower loads to failure compared with the PU and U regions in the same animal. However, only 2 samples of each region with intact linea alba were tested, precluding statistical analysis and representing a limitation of our study. All control samples failed distant to the linea alba, highlighting the high intrinsic resistance to failure of intact linea alba. In our study, the thickness of the linea alba decreased gradually from cranial to caudal in all cats, being thinner and biomechanically weaker in the POU region than in the PU and U regions. In a human cadaveric study, fascial thickness contributed 32% of variability in suture pull‐out force in the linea alba.^12^ In our study, thickness of linea alba accounted for 35.4% of variability in load to failure, suggesting that linea thickness plays a role in strength of celiotomy closure in cats.

No difference in breaking strength of celiotomy samples was observed between the 2 suture materials (polydioxanone and polyglactin 910) used in our study. There is no experimental or clinical evidence of a continuous suture pattern increasing the risk of wound disruption.^2^ Continuous suture patterns distribute tension along the entire incisional length, decreasing strain at individual suture bites.^13^ Simple continuous closure of the ventral abdominal wall is well accepted in dogs and cats,^4^, ^14^ likely because it is faster than simple interrupted closure^4^ and does not increase the risk of wound disruption.^3^ We did not detect differences in breaking strengths for celiotomies closed with simple continuous fascia‐only or full‐thickness bites in cats. To the best of our knowledge, fascia‐only closure has not been previously reported in the domestic cat. The external leaf of the rectus abdominis sheath has been shown to serve as the primary strength‐holding layer for ventral paramedian incision closure in dogs. This concept is based on a lack of difference in biomechanical properties of fascia‐only and full‐thickness closure, which was attributed to incorporating the external leaf in both groups.^9^ In our study, fascia‐only closure is biomechanically equivalent to full‐thickness closure in the cat, likely for the same reason. Fascia‐only closure could potentially reduce postoperative incisional swelling and pain in feline patients after abdominal surgery. Inclusion of muscle in sutured wounds is considered to increase pain during the postoperative period.^2^

SL:WL ≥4:1 has been recommended for median celiotomy closure in humans,^15^, ^16^, ^17^ given that incisional herniation has been found more common in wounds closed with SL:WL <4:1.^16^, ^17^ A recent trend in human median abdominal closure favors small bites over large bites while observing this minimal SL:WL of 4:1.^18^, ^19^ Our study was designed to provide SL:WL >4:1. We arbitrarily chose 2‐mm and 5‐mm bites, representing small and large bite closure, respectively. Advantages of small bite closure include reduced incidence of surgical site infection, better distribution of tension across the celiotomy closure, and reduced incidence of incisional herniation.^17^, ^18^, ^19^ Large SBSI may cause the suture to cut through or compress soft tissues when tension is applied, making the stitch loosen and allowing wound edges to separate.^17^, ^18^ We observed this pattern of failure most commonly in our 5 × 5 SBSI celiotomy groups. No differences in breaking strength were observed in our celiotomy samples comparing the 2 SBSI used; however, evaluation of influence of SBSI in multivariable analysis and mean load to failure between suture material/SBSI samples approached significance. A larger sample could have revealed significant differences between the 2 SBSI used.

In humans, sutures engaging the transition zone between linea alba and rectus sheath markedly increase strength of celiotomy closure.^20^ In small animals, this transition zone has not been described, and the distance between the linea alba and the site of needle penetration during celiotomy closure is rarely reported^16^ or is based on recommendations rather than biomechanical testing.^4^, ^8^, ^21^ We designed our study to engage the transition zone in all celiotomies, measuring suture bites from the lateral edge of the linea alba. In this setting, no difference was detected between closures incorporating small or large bites.

Muscle/fascial tearing was the most common type of failure observed for all celiotomy samples in our study, without any knot failure. Instead, cracks initiated by stitches at sites of needle penetration extended longitudinally toward the celiotomy. The biomechanical significance of this type of failure is unknown but has been previously described in an equine bursting model in which fascial failure was the main failure mode observed for the 2 suture intervals used.^22^ The type of needle used could have influenced this type of failure. However, we used a fine, atraumatic needle of consistent size. It is unknown if different needle type or size could produce different results.

Tensile testing of connective tissue is logistically difficult because low friction at the grip–tissue interface often leads to slippage prior to failure.^23^ Cryoclamps have been used for tensile testing of the linea alba of horses^24^ because they allow better gripping of tissue and increase friction between tissue and grips. The custom‐made clamps used in our study were previously used in a biomechanical study of muscles in other species.^25^ No slippage was reported in the Chism et al^25^ study with the use of dumbbell‐shaped specimens, as recommended for tensile testing of elastomers^10^ and cheese grater clamps. No slippage at tissue–grip interface was observed in our study.

Fresh feline cadavers were not available for our study. However, we do not believe that freezing affected our results because of its lack of influence on the breaking strength of the canine linea alba.^26^ In the Rath et al^26^ study, samples of abdominal wall were frozen after harvest, with muscle contraction caused by freezing artifact and more difficulties to secure samples in grips reported. In our study, whole cadavers were frozen and thawed intact prior to sampling and testing. We did not observe changes in the abdominal wall or experience difficulty in securing samples in grips. After thawing, samples were collected and preserved in a saline bath under refrigeration. This protocol, reported in another study with samples kept up to 24 hours,^27^ yields a state of mechanical stability making the samples useful for analysis. Nonetheless, limitations of our study remain inherent to its ex vivo design. Suture degradation, surgical site infection, prolonged inflammation, suture failure during cyclic and repetitive tension, and other patient‐related factors that contribute to incisional hernia in vivo following ventral midline celiotomy closure^2^, ^3^, ^13^, ^16^, ^17^, ^19^ could not be evaluated in our ex vivo study. We also tested discrete segments of ventral abdominal wall, providing an idea of the dynamic properties of the 3 regions tested and their biomechanical properties but making extrapolations of results to the entire ventral abdominal wall impossible. Another limitation relates to the method of tensile testing. We used uniaxial loading in a single test to failure with continuous distraction. This design differs from the clinical scenario, in which strains are cyclic and dependent on the animal's movement during the postoperative period. Cyclic strain on an incision may result in different results compared with continuous distraction. We chose a low speed of distraction (0.5 mm/second, 30 mm/minute) for our study because of the viscoelastic nature of soft tissue specimens. In previous studies, a different speed of distraction was used,^12^, ^24^, ^26^ making direct comparisons of results impossible. In addition, 2 types of forces act on the ventral abdominal wall, intra‐abdominal pressure acting on the deep aspect and linear traction exerted by flank muscles.^27^ We evaluated elasticity and elongation in discrete segments to mimic linear traction of flank muscles. However, the interaction of surrounding muscles of the entire ventral abdominal wall was not evaluated. Different results may be obtained by testing bursting strength of the abdomen and celiotomy closure techniques and by evaluating tensile forces in the entire ventral abdominal wall. A combination of tensile and bursting tests may be more representative of forces that celiotomy closure encounters after surgery in vivo. Another limitation is the small number of specimens used and samples tested for variables used in the statistical model. Due to randomization, the number of samples from each ROI and the specimen where the samples were obtained varied among groups. A minimum of 20 samples per ROI (equivalent to 20 cats) would have been required to obtain 80% statistical power. The coefficient of variation between the different specimens tested represents another limitation of the study. Biological tissue has a high degree of natural variation, both interindividual and intraindividual. A large number of tests and a uniform sample population are required to extrapolate results to the clinical scenario. Although ex vivo studies can aid in determining adequate suture techniques for celiotomy closure in domestic cats, prospective studies evaluating the 2 SBSI that we used or other SBSI combinations and complications associated with their use, including incidence of incisional herniation, would be required to determine optimal technique for celiotomy closure in this species.

In conclusion, the POU region of ventral abdominal wall in domestic cats is biomechanically weaker than the PU and U regions under uniaxial loading in this model. Considering the biomechanical results and types of failure observed in our study, we favor simple continuous closure using 2 × 2‐mm SBSI and including the external leaf of rectus abdominis muscle sheath in cats. However, additional biomechanical and clinical studies are warranted before specific celiotomy closure recommendations can be made.

## CONFLICT OF INTEREST

The authors declare no conflicts of interest related to this report.
